# Statin use and incident cardiovascular events in renal transplant recipients

**DOI:** 10.1111/eci.13594

**Published:** 2021-05-27

**Authors:** Josephine L. C. Anderson, Markus van der Giet, Antonio W. Gomes Neto, Stephan J. L. Bakker, Uwe J. F. Tietge

**Affiliations:** ^1^ Department of Internal Medicine University Medical Center Groningen University of Groningen Groningen The Netherlands; ^2^ Medizinische Klinik für Nephrologie und Internistische Intensivtherapie Charité – Universitätsmedizin Berlin Berlin Germany; ^3^ Division of Clinical Chemistry Department of Laboratory Medicine Karolinska Institutet Stockholm Sweden; ^4^ Clinical Chemistry Karolinska University Laboratory Karolinska University Hospital Stockholm Sweden

**Keywords:** cardiovascular disease, cyclosporine, pharmacological interaction, renal transplantation, statins

## Abstract

**Background:**

Statins achieve potent LDL lowering in the general population leading to a significant cardiovascular (CV) risk reduction. In renal transplant recipients (RTR) statins are included in treatment guidelines, however, conclusive evidence of improved cardiovascular outcomes has not been uniformly provided and concerns have been raised about simultaneous use of statins and the immunosuppressant cyclosporine. This study aimed to elucidate the effect of statins on a compound CV endpoint, comprised of ischaemic CV events and CV mortality in RTR, with subgroup analysis focussing on cyclosporine users.

**Method:**

622 included RTR (follow‐up 5.4 years) were matched based on propensity scores and dichotomized by statin use. Survival analysis was conducted.

**Results:**

Cox regression showed that statin use was not significantly associated with the compound CV endpoint in a fully adjusted model (HR = 0.81, 95% CI = 0.53‐1.24, *P* = .33). Subgroup analyses in RTR using cyclosporine revealed a strong positive association of statin use with the CV compound outcome in a fully adjusted model (HR = 6.60, 95% CI 1.75‐24.9, *P* = .005). Furthermore, statin use was positively correlated with cyclosporine trough levels (correlation coefficient 0.11, *P* = .04).

**Conclusion:**

In conclusion, statin use does not significantly decrease incident CV events in an overall RTR cohort, but is independently associated with CV‐specific mortality and events in cyclosporine using RTR, possibly due to a bilateral pharmacological interaction.

## 1 INTRODUCTION

HMG‐CoA reductase inhibitors, commonly referred to as statins, are among the world's most widely prescribed medications. Atorvastatin became the best‐selling pharmaceutical in history in 2003, under the brand name Lipitor, with a yearly revenue of above 10 billion US dollars.^1^, ^2^ Statins potently reduce circulating low‐density lipoprotein cholesterol (LDL‐C) levels, a prime risk factor for atherosclerotic cardiovascular disease (CVD). Statins are thus a mainstay of anti‐atherosclerotic therapy, with proven efficacy in primary, as well as secondary, CVD prevention.^3^ Overall, the introduction of statins has resulted in a 15%‐30% decrease in the incidence of cardiovascular mortality in the general population.^4^, ^5^, ^6^

However, in selected high‐risk populations most in need of CVD risk management the effect of statins is less evident. End‐stage renal disease (ESRD) patients, for example, suffer from an age adjusted 30‐fold increase in CVD mortality that is not substantially reduced by statins.^7^ Another patient population particularly vulnerable for development of CVD are renal transplant recipients (RTR). Due to the increasing success of renal transplantations, the number of RTR is ever increasing, in several countries even surpassing that of patients on maintenance dialysis.^8^ However, RTR still suffer an exceptionally high, but (patho)physiologically still poorly understood, mortality burden.^9^ The biggest threat to this patient group is a vast increase in CVD mortality, translating to a 4‐6 times higher age‐adjusted risk compared to the general population.^10^, ^11^ Also in RTR, the effect of statins is uncertain since evidence is sparse and has limitations, such as incomplete follow‐up, leading to low quality of evidence.^12^

Furthermore, concern has been raised about the combination of statins with immunosuppressive regimen. In particular, it has been suggested that simultaneous use of statins and the immunosuppressive drug cyclosporine leads to an increased unbound fraction of serum cyclosporine, with imaginable adverse consequences.^13^

Nonetheless, statins were included in treatment guidelines for RTR, due to the fact that ‘it was assumed that similar treatment efficacy to that reported in the general population would be found if the trials were carried out in kidney transplant patients.’^14^ Studies directly proving this assumption are thus far not available. The aim of this study is therefore to assess the effect of statins on incident CVD in a well‐characterized cohort of RTR, with subgroup analysis in cyclosporine using RTR.

## 2 METHODS

### 2.1 Patient population

**FIGURE 1:**
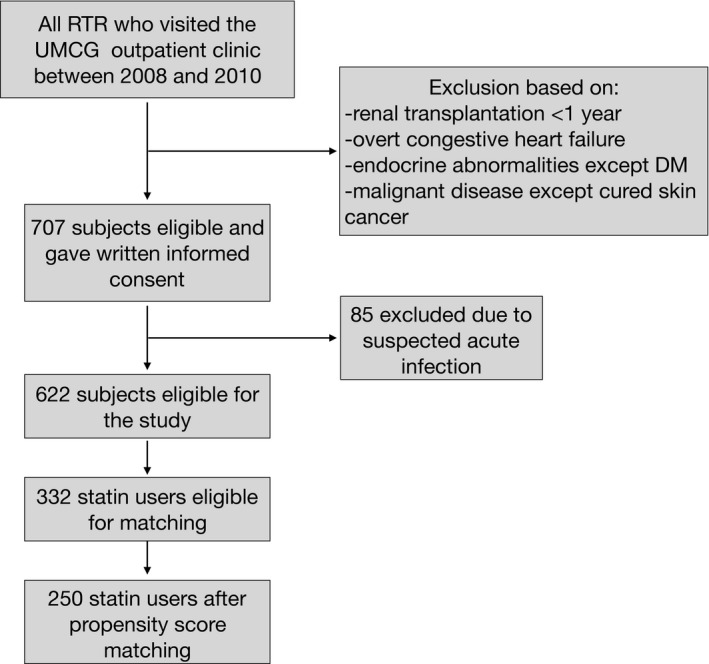
Inclusion of renal transplant recipients. RTR, renal transplant recipients; UMCG, University Medical Centre Groningen; DM, diabetes mellitus

For this follow‐up study, all renal transplant recipients who visited the University Medical Centre Groningen (UMCG) outpatient clinic between November 2008 and March 2011, with a functioning allograft for at least 1 year, were invited to participate. Patients diagnosed with overt congestive heart failure, endocrine abnormalities except diabetes or malignant disease other than cured skin cancer were not eligible for inclusion. Of the 707 patients that gave written informed consent, 85 were excluded due to a suspected acute infection, as indicated by a CRP value of >15mg/L (Figure 1). The remaining 622 patients were followed for a median of 5.4 years (25th–75th interquartile range (IQR) 4.9‐6.0 years), and no patients were lost during follow‐up. All relevant patient characteristics were obtained from the ‘Groningen Renal Transplant Database’. More detailed definitions of the characteristics of the database, patients’ baseline characteristics and routine laboratory methods used have been previously reported.^15^ The study protocol was approved by the University Medical Centre Groningen Institutional Review Board (METc 2008/186) and is in accordance with the Declaration of Helsinki. The ‘TransplantLines Food and Nutrition Biobank and Cohort Study’ is registered at clinicaltrials.gov as NCT02811835.

### 2.2 Immunosuppressive medication

RTRs all received standard immunosuppressive therapy. Standard immunosuppression consisted of the following: cyclosporine (target trough levels 175‐200 mg/L in the first 3 months, 100 mg/L thereafter) and prednisolone (starting with 20 mg/d and tapering to 10 mg/d) from 1989 to 1996. In 1997, mycophenolate mofetil (2 g/d) was added to the standard immunosuppressive regimen. In 2012, cyclosporine was replaced by tacrolimus, and RTRs continued triple‐immunosuppressive therapy with prednisolone (20 mg/d, tapering to 5 mg/d), tacrolimus (target trough levels 8‐12 μg/L in the first 3 months, 6‐10 μg/L until month 6 and 4‐6 μg/L from 6 months onward) and mycophenolate mofetil (starting with 2 g/d, tapering to 1 g/d). A cyclosporine‐based regime is advised if side effects of tacrolimus occur and in post‐transplantation DM patients.

### 2.3 Endpoint

The main outcome measure in this study was the use of any type of statin at the time of inclusion. The primary endpoint of this study was a compound CVD endpoint, consisting of the first occurrence of an ischaemic CV event or CVD death. The following events were considered ischaemic in nature and were included: myocardial infarction (MI), angina pectoris, coronary artery bypass grafting (CABG), percutaneous transluminal coronary angioplasty (PTCA) and ischaemic cerebral infarction. Patients were censored for non‐CV causes of mortality. Cause of death was obtained by linking the number of the death certificate to the primary cause of death as coded by a physician from the Central Bureau of Statistics. Cause of death was coded according to the International Classification of Disease, 9th revision (ICD‐9). CVD mortality was defined as the principal cause of death being cardiovascular in origin, namely ICD‐9 codes 410‐447.

### 2.4 Measurements and definitions

Information regarding medication was extracted from patients’ medical records. Blood samples were drawn after a 8‐12 hours fasting period, prior to medication intake. Serum high‐sensitivity C‐reactive protein (hsCRP), glycated haemoglobin (HbA1C), triglycerides, total cholesterol, LDL‐C and high‐density lipoprotein cholesterol (HDL‐C) were measured using routine laboratory methods. A modified version of the Jaffé method was used to determine serum creatinine (MEGA AU 510; Merck Diagnostica). All participants were instructed to collect a 24‐hours urine sample the day before their visit to the outpatient clinic. Total urinary protein concentration was determined using the Biuret reaction (MEGA AU 150, Merck Diagnostica).

Diabetes was defined as use of anti‐diabetic medication, fasting plasma glucose ≥7.0 mmol/L or HbA1C higher than 6.5%.^16^ Proteinuria was present when urinary protein excretion was ≥0.5 g/24 h. A history of an atherosclerotic CV event was defined as the occurrence of an MI, angina pectoris, CABG, PTCA or ischaemic cerebral infarction.

### 2.5 Study design

To address potential confounders for the primary prespecified analysis, we used propensity score matching to compare the CVD compound endpoint between subjects receiving statin therapy and those who did not. A logistic regression was fitted for use of statins, including variables that, based on literature, are related to the outcome. This included patient demographics (age, sex), lifestyle factors (smoking, alcohol use), lipid biomarkers (HDL‐C, LDL‐C, triglycerides), kidney function parameters (serum creatinine, urinary protein excretion), CVD risk factors (history of MI, CVA or coronary intervention, systolic blood pressure, number of antihypertensives), medication use (prednisolone dose, use of proliferation inhibitors, use of tacrolimus, use of cyclosporine) and co‐morbidities (primary renal disease, hsCRP, dialysis time, diabetes mellitus (DM), glucose levels, metabolic syndrome).^17^ Propensity scores were obtained from the outcome of the logistic regression. Statin users were matched to nonstatin users by one to one nearest‐neighbour matching with replacement, meaning that a control subject could be used in multiple case‐control pairs, allowing for more optimal matching.^18^ Quality of matching was graphically evaluated (supplemental Figure 1) and the reduction of bias assessed using a t test for equality of means, the standardized percentage bias and the variance ratio (supplemental Table 1). Survival analysis was conducted with weighing for the propensity score.

### 2.6 Statistical analysis

Differences in baseline characteristics were assessed between statin users and nonstatin users in the unmatched entire cohort, as well as in the propensity score‐matched subset. Continuous, normally distributed variables are presented as mean (±SD) and differences tested with a one‐way ANOVA. Continuous variables with a skewed distribution are given as median (25th, 75th percentile) and differences tested by Mann‐Whitney U test. Categorical data are summarized by n (%) and differences tested by the chi‐squared test.

**FIGURE 2:**
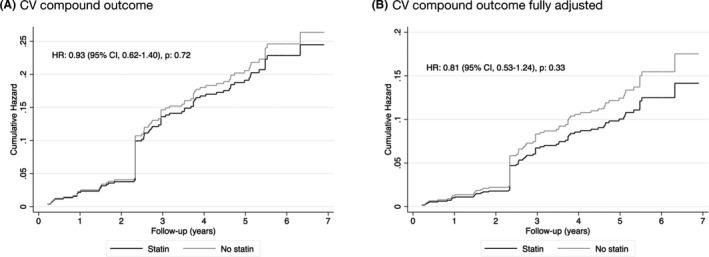
Comparison of the Cumulative Hazard of the cardiovascular (CV) compound endpoint of statin use versus no statin use in the propensity‐matched cohort. Hazard ratios were obtained using weighted Cox proportional hazard regressions. Fully adjusted models were adjusted for age, metabolic syndrome, total cholesterol, LDL‐C, HbA1c, use of cyclosporine and living kidney donors. CV, cardiovascular; HR, hazard ratio; 95% CI, 95% confidence interval

**FIGURE 3:**
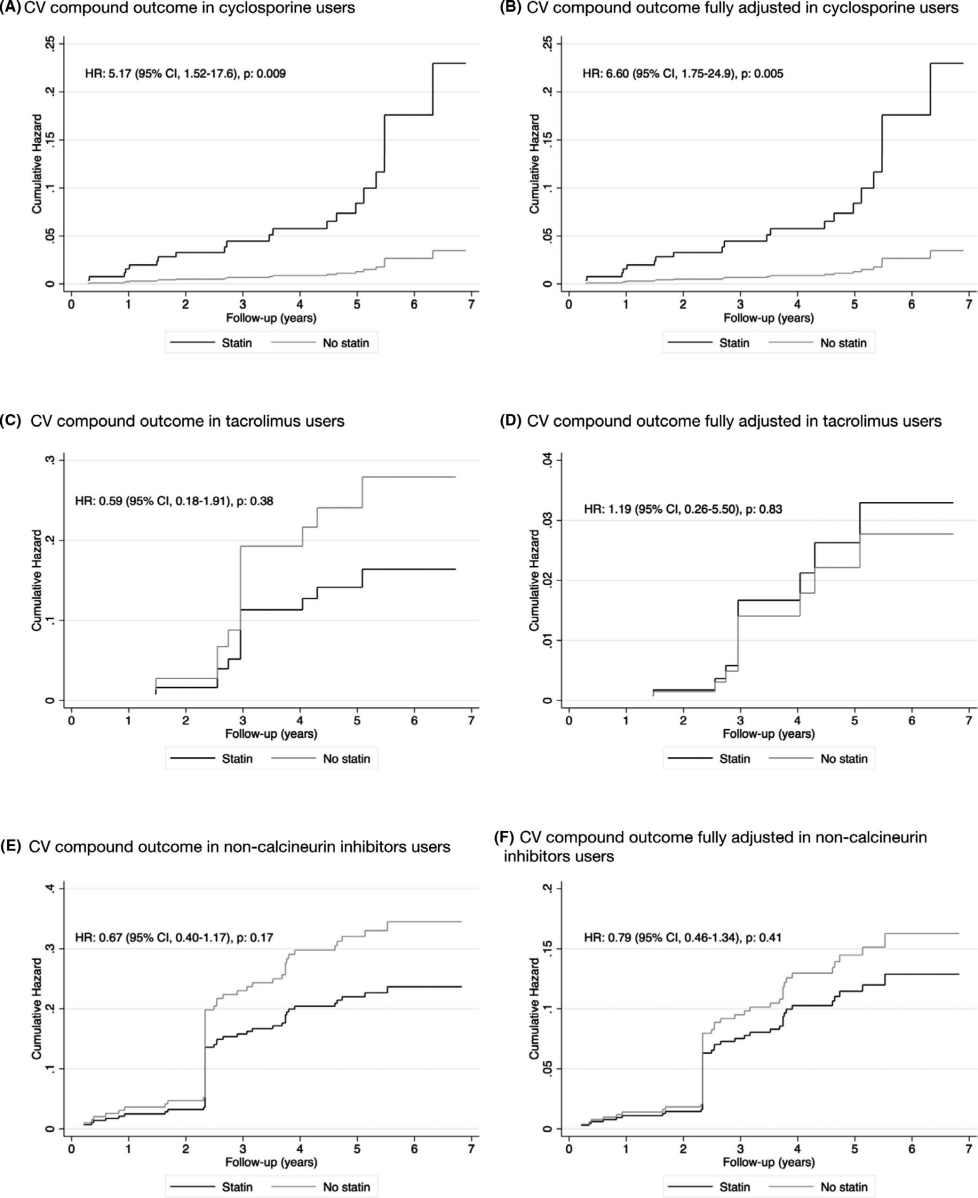
Comparison of the cumulative hazard of the cardiovascular compound endpoint of statin use versus no statin use by cyclosporine use, tacrolimus use and no use of calcineurin inhibitors in the propensity‐matched cohort. Hazard ratios were obtained using weighted Cox proportional hazard regressions. Fully adjusted models were adjusted for age, metabolic syndrome, total cholesterol, LDL‐C, HbA1c, use of cyclosporine and living kidney donors. CV, cardiovascular; HR, hazard ratio; 95% CI, 95% confidence interval

Cox proportional hazards regression was performed in the propensity‐matched cohort, using weighted estimations based on the frequency with which a single observation was used as a match. Cumulative hazards were computed for the endpoints. Due to the fact that matching was done with replacement and the analysis weighted based on this, one participant counts as a control subject for a variable number of times. This accounts for sudden increases in the cumulative hazard rate, as seen in Figure 2 and Figure 3. Analyses were performed both crude, as well as with further adjustment for covariates for which balance was not achieved with matching, indicated by significant differences between groups. This included age, metabolic syndrome, total cholesterol, LDL‐C, HbA1c, use of cyclosporine and living kidney donors. Subgroup analysis was conducted in subjects receiving cyclosporine treatment, subjects receiving tacrolimus treatment and subjects that did not receive treatment with a calcineurin inhibitor. The proportional hazards assumption was tested using log‐log graphs and was found not to be violated.

A *P*‐value of <.05 was considered statistically significant. All statistical analyses were performed using STATA version 15 (2017, StataCorp). Reporting of the study conforms to broad EQUATOR guidelines.^19^

## 3 RESULTS

### 3.1 Baseline demographic characteristics

A total of 707 subjects from the ‘TransplantLines Food and Nutrition Biobank and Cohort Study’ were assessed for eligibility. After exclusion due to suspected acute infection, as determined by a hsCRP >15 mg/L, 622 subjects were eligible for inclusion in the cohort (Figure 1). The matching procedure matched 250 statin users to 90 nonstatin users. Due to matching with replacement, 250 case‐control pairs were matched. Standardized percentage bias and the variance ratio are shown in Supplemental Table 1.

Of the 622 RTR originally included in the study, 332 (53%) RTR received statins. In the propensity‐matched cohort 250 subjects used statins, of which 163 (48% of statin users) used atorvastatin, 52 (15% of statin users) used simvastatin, 13 (4% of statin users) used fluvastatin, 12 used rosuvastatin (4% of statin users), and 11 (3% of statin users) used pravastatin.

Baseline characteristics for patients receiving statins and those not receiving statins in the entire cohort are summarized in Table 1. As expected, significant differences between the groups were found for known CVD risk factors, components of the metabolic syndrome, cholesterol measures, including significantly lower LDL‐C levels in statin users, history of ischaemic CV events, use of anti‐diabetic medication and markers of insulin resistance. After matching, considerable improvement in balance was achieved for most patient characteristics (Table 2). Age, presence of metabolic syndrome, total cholesterol, HbA1c and living kidney donor remained significantly higher in the statin group, while LDL‐C remained significantly lower.

**TABLE 1:** Baseline characteristics according to use of statins in the entire unmatched cohort (n = 622)

Characteristic	No use of statins (n = 290)	Use of statins (n = 332)	*P*‐value
Recipient demographics			
Age, years	50.2 (39.6, 61.7)	57.1 (49.0, 64.0)	<.001
Male gender, n (%)	167 (58%)	189 (57%)	.87
Current smoking, n (%)	34 (12%)	39 (13%)	.93
Former smoking, n (%)	110 (40%)	160 (52%)	.004
Never smoking, n (%)	131 (48%)	110 (36%)	.003
Metabolic syndrome, n (%)	126 (43%)	260 (78%)	<.001
Body composition			
BMI	26.1 ± 4.9	27.1 ± 4.7	.008
Lipid profile			
Total cholesterol, mmol/L	5.4 ± 1.1	4.9 ± 1.1	<.001
LDL cholesterol, mmol/L	3.3 ± 0.9	2.7 ± 0.9	<.001
HDL cholesterol, mmol/L	1.4 ± 0.5	1.4 + 0.5	.92
Triglycerides, mmol/L	1.6 (1.2, 2.2)	1.7 (1.3, 2.4)	.003
Cardiovascular disease history			
History of MI, CVA or coronary intervention, n (%)	29 (10%)	62 (19%)	.003
Blood pressure			
Systolic blood pressure, mmHg	134.8 ± 16.5	137.0 ± 18.3	.09
Diastolic blood pressure, mmHg	83.0 ± 11.7	82.1 ± 10.4	.31
Use of ACE inhibitors, n (%)	87 (30%)	117 (35%)	.17
Use of *β*‐blockers, n (%)	160 (55%)	225 (68%)	.001
Use of diuretics, n (%)	96 (33%)	150 (45%)	0002
Number of antihypertensive drugs, n	2 (1, 2)	2 (1, 3)	<.001
Glucose homeostasis			
Glucose, mmol/L	5.2 (4.7, 5.8)	5.3 (4.8, 6.2)	.016
HbA1c, %	5.6 (5.4, 6)	5.9 (5.6, 6.4)	<.001
Diabetes mellitus, n (%)	50 (17%)	94 (28%)	.001
Use of anti‐diabetic drugs, n (%)	28 (10%)	66 (20%)	<.001
Use of insulin, n (%)	17 (6%)	39 (12%)	.011
Inflammation			
hsCRP, mg/L	1.7 (0.8, 5.1)	1.5 (0.6, 4.0)	.05
Donor demographics			
Age, years	47 (33, 55)	46 (32, 54)	.85
Male gender, n (%)	148 (52%)	170 (52%)	1.0
Living kidney donor, n (%)	108 (37%)	112 (34%)	.36
(Pre)transplant history			
Dialysis time, months	25 (7, 48)	28 (13, 54)	.03
HLA mismatches	2 (1, 3)	2 (1, 3)	.63
Time between tx and baseline, years	5.0 (1.4, 10.1)	5.6 (2.1, 12.4)	.11
Primary renal disease			
Primary glomerular disease, n (%)	79 (27%)	95 (29%)	.70
Glomerulonephritis, n (%)	19 (7%)	30 (9%)	.25
Tubulointerstitial disease, n (%)	37 (13%)	33 (10%)	.27
Polycystic renal disease, n (%)	64 (22%)	63 (19%)	.03
Dysplasia and hypoplasia, n (%)	13 (4%)	13 (4%)	.72
Renovascular disease, n (%)	18 (6%)	20 (6%)	.92
Diabetic nephropathy, n (%)	12 (4%)	20 (6%)	.29
Other or unknown cause, n (%)	48 (17%)	58 (17%)	.76
Immunosuppressive medication			
Daily prednisolone dose, mg	10 (7.5, 10)	10 (7.5, 10)	.49
Calcineurin inhibitors, n (%)	172 (59%)	190 (57%)	.60
Tacrolimus, n (%)	67 (23%)	50 (15%)	.01
Cyclosporine, n (%)	106 (37%)	140 (42%)	.15
Proliferation inhibitors, n (%)	242 (83%)	276 (83%)	.01
Azathioprine, n (%)	42 (15%)	59 (18%)	.27
Mycophenolate mofetil, n (%)	200 (69%)	217 (65%)	.34
Renal allograft function			
Serum creatinine, μmol/L	122 (118, 129)	123 (99, 160)	.74
Urinary protein excretion, g/24 h	0.2 (0, 0.4)	0.2 (0, 0.4)	.33

Note

Normally distributed continuous variables are presented as mean ± SD, and differences were tested with one‐way ANOVA. Continuous variables with a skewed distribution are presented as median (25th, 75th percentile), and differences were tested by Mann‐Whitney test. Categorical data are summarized by n (%), and differences were tested by chi‐squared test.

Abbreviations: ACE, angiotensin‐converting enzyme; BMI, body mass index; HbA1C, glycated haemoglobin; HDL, high‐density lipoprotein; HLA, human leucocyte antigen; hsCRP, high‐sensitivity C‐reactive protein; LDL, low‐density lipoprotein; MI, myocardial infarction, CVA, cerebrovascular event; tx, transplantation.

**TABLE 2:** Baseline characteristics according to use of statins in the propensity score‐matched cohort (n = 340)

Characteristic	No use of statins (n = 90)	Use of statins (n = 250)	*P*‐value
Recipient demographics			
Age, years	53.1 (44.5, 64.3)	56.7 (49.4, 63.6)	.036
Male gender, n (%)	41 (45%)	105 (42%)	.56
Current smoking, n (%)	13 (14%)	33 (13%)	.77
Former smoking, n (%)	43 (48%)	129 (52%)	.53
Never smoking, n (%)	34 (38%)	88 (35%)	0.003
Metabolic syndrome, n (%)	59 (66%)	191 (76%)	.046
Body composition			
BMI	26.2 ± 4.5	26.9 ± 4.6	.22
Lipid Profile			
Total cholesterol, mmol/L	5.3 ± 1.2	5.0 ± 1.1	.011
LDL cholesterol, mmol/L	3.1 ± 1.0	2.8 ± 0.9	.001
HDL cholesterol, mmol/L	1.4 ± 0.5	1.4 ± 0.5	.92
Triglycerides, mmol/L	1.9 (1.3, 2.6)	1.7 (1.3, 2.5)	.70
Cardiovascular disease history			
History of MI, CVA or coronary intervention, n (%)	14 (16%)	45 (18%)	.60
Blood pressure			
Systolic blood pressure, mmHg	135.0 ± 15.4	137.0 ± 18.1	.34
Diastolic blood pressure, mmHg	83.1 ± 10.9	82.4 ± 10.8	.59
Use of ACE inhibitors, n (%)	29 (32%)	91 (36%)	.48
Use of *β*‐blockers, n (%)	61 (68%)	169 (68%)	.98
Use of diuretics, n (%)	41 (46%)	112 (45%)	.90
Number of antihypertensive drugs, n	2.0 (1.0, 3.0)	2.0 (1.0, 3.0)	.56
Glucose homeostasis			
Glucose, mmol/L	5.2 (4.7, 5.9)	5.3 (4.7, 6.0)	.58
HbA1c, %	5.7 (5.5, 6.1)	5.9 (5.6, 6.3)	.016
Diabetes mellitus, n (%)	19 (21%)	63 (25%)	.44
Use of anti‐diabetic drugs, n (%)	13 (14%)	44 (18%)	.49
Use of insulin, n (%)	6 (7%)	26 (10%)	.30
Inflammation			
hsCRP, mg/L	1.4 (0.8, 2.9)	1.4 (0.6, 3.2)	.66
Donor demographics			
Age, years	44.5 (29.0, 55.0)	46.0 (34.0, 54.0)	.46
Male gender, n (%)	37 (41%)	136 (54%)	.031
Living kidney donor, n (%)	19 (21%)	90 (36%)	.009
(Pre)transplant history			
Dialysis time, months	30.0 (9.0, 50.0)	25.5 (10.0, 52.0)	.80
HLA mismatches	2.0 (1.0, 3.0)	2.0 (1.0, 3.0)	.15
Time between tx and baseline, years	6.1 (2.5, 11.4)	5.4 (2.2, 12.5)	.76
Primary renal disease			
Primary glomerular disease, n (%)	26 (29%)	79 (32%)	.63
Glomerulonephritis, n (%)	5 (6%)	16 (6.4%)	.78
Tubulointerstitial disease, n (%)	13 (14%)	24 (10%)	.21
Polycystic renal disease, n (%)	17 (19%)	48 (19%)	.95
Dysplasia and hypoplasia, n (%)	3 (3%)	8 (3%)	.95
Renovascular disease, n (%)	4 (4%)	18 (7%)	.36
Diabetic nephropathy, n (%)	6 (7%)	13 (5%)	.60
Other or unknown cause, n (%)	16 (18%)	44 (18%)	.97
Immunosuppressive medication			
Daily prednisolone dose, mg	10.0 (7.5, 10.0)	10.0 (7.5, 10.0)	.99
Calcineurin inhibitors, n (%)	50 (56%)	137 (55%)	.95
Tacrolimus, n (%)	19 (21%)	32 (13%)	.21
Cyclosporine, n (%)	31 (34%)	105 (42%)	.058
Proliferation inhibitors, n (%)	71 (79%)	207 (83%)	.41
Azathioprine, n (%)	10 (11%)	43 (17%)	.17
Mycophenolate mofetil, n (%)	61 (68%)	164 (66%)	.71
Renal allograft function			
Serum creatinine, μmol/L	123.5 (97.0, 166.0)	122.5 (100.0, 156.0)	.93
Urinary protein excretion, g/24 h	0.2 (0.0, 0.4)	0.2 (0.0, 0.3)	.75

Note

Normally distributed continuous variables are presented as mean ± SD, and differences were tested with one‐way ANOVA. Continuous variables with a skewed distribution are presented as median (25th, 75th percentile), and differences were tested by Mann‐Whitney test. Categorical data are summarized by n (%), and differences were tested by chi‐squared test.

Abbreviations: ACE, angiotensin‐converting enzyme; BMI, body mass index; HbA1C, glycated haemoglobin; HDL, high‐density lipoprotein; HLA, human leucocyte antigen; hsCRP, high‐sensitivity C‐reactive protein; LDL, low‐density lipoprotein; MI, myocardial infarction, CVA, cerebrovascular event; tx, transplantation.

### 3.2 Time to event analysis

The CV compound endpoint was reached in 56 (16.5%) of the included RTR in the propensity‐matched cohort, of which 44 received statin therapy and 12 did not (*P* = .35). Cumulative hazard ratios were computed for the CV compound endpoint. In a crude analysis, the use of statins was not significantly associated with the endpoint (HR = 0.93, 95% confidence interval [CI] = 0.62‐1.40, *P* = .72) (Figure 2A). Adjustment for variables for which balance was not achieved with matching, namely age, metabolic syndrome, total cholesterol, HbA1c, LDL‐C and living kidney donation, did not impact this association (HR = 0.81, 95% CI = 0.53‐1.24, *P* = .33).

Previous work suggested a potential interaction between statins and cyclosporin.^13^

Indeed, in our cohort a significant correlation existed between the use of statins and blood levels of cyclosporine, with a correlation coefficient of 0.11 (*P* = .04), meaning that use of statins accounts for 11% of the variability of cyclosporine levels.

In order to better understand the effect of simultaneous use of statins and cyclosporine, subgroup analysis was performed in RTR receiving cyclosporine treatment, those receiving tacrolimus treatment and those who did not receive treatment with a calcineurin inhibitor. Interestingly, use of statins was strongly positively associated with the CV compound endpoint in cyclosporine users (HR = 5.17, 95% CI = 1.52‐17.6, *P* = .009), translating to a 5‐fold increased risk in reaching the endpoint in statin users. Again, a fully adjusted analysis was performed, which did not substantially alter the association (HR = 6.6, 95% CIs 1.75‐24.9, *P* = .005). On the other hand, in RTR receiving tacrolimus therapy (HR = 1.19, 95% CI = 0.26‐5.50, *P* = .83) and in RTR not receiving calcineurin therapy (HR = 0.79, 95% CI = 0.46‐1.34, *P* = .41) use of statins had no effect on the CV compound endpoint.

## 4 DISCUSSION

The results of this study demonstrate that use of statins is not associated with CV events or mortality in renal transplant recipients meaning that no overall protective effect of statin therapy was discernible in this patient group. In fact, use of statins in cyclosporine treated patients had a strong positive association with incident CV events and mortality.

Statins are a standard treatment modality in RTR, with the goal of reducing CVD risk through lowering of LDL‐C. The topic is addressed in the most recent version of the guidelines from the Lipid Guideline Development Work Group of the Kidney Disease: Improving Global Outcomes (KDIGO) organization about lipid management in chronic kidney disease (CKD).^20^ By definition, RTR are considered to have CKD, but are additionally also specifically referred to in the guidelines, which state that all RTR, irrespective of age and LDL‐C levels, should receive statin therapy.^20^ The rationale behind this recommendation is based on a single randomized trial, namely the Assessment of LEscol in Renal Transplantation (ALERT) trial. ALERT investigated the effect of statins in 2102 RTR, with an age range of 30‐75 years, who were followed for a mean of 5.1 years.^21^ However, no significant association was seen with the primary endpoint, which was the first occurrence of a major adverse cardiac event, defined as cardiac death, nonfatal MI or coronary artery bypass.^21^ A significant risk reduction was found in secondary endpoints, and however, the authors themselves stated that the results should be interpreted with caution due to the absence of a significant primary endpoint and lack of correction for multiple comparisons.^21^ A later post hoc subgroup analysis reached the conclusion that cardiac death and nonfatal myocardial infarction were prevented by statins in RTR.^22^

With respect to our present study, several possible explanations exist for not only the lack of efficacy of statins in RTR, but the consequent increase in the CVD endpoint specifically in cyclosporine users. These include i.) a different underlying pathophysiological basis of CVD in RTR, ii.) reduced efficacy of statins due to reduced kidney function and iii.) concurrent use of immunosuppressive medication.

LDL‐C is widely applied in estimating future risk of CVD in the general population, due to its causal contribution to atherosclerotic disease, which provides the rationale for the use of statins in RTR. However, in RTR traditional CVD risk factors, including LDL‐C, do not uniformly predict CVD mortality to the same extent as in the general population,^23^ therefore suggesting that a different pathophysiological mechanism contributes to CVD. Furthermore, atherosclerosis might be less frequently the underlying cause of CVD mortality in CKD patients, and consequently as well in RTR, with a higher proportion of sudden death, arrhythmia and heart failure.^10^, ^24^ This potentially explains why LDL‐C lowering in the form of statins does not decrease CVD mortality in patients with impaired renal function.^7^

Furthermore, RTR have a lower kidney function due to various factors, including ischaemic and reperfusion injuries, revascularization and nephrotoxicity of immunosuppressants.^25^ In addition, most RTR have a history of ESRD and haemodialysis. CKD has previously been linked to a reduced efficacy of statins.^26^ The efficacy seems to be further reduced with deteriorating renal function. Two trials have shown that in haemodialysis patients statins do not have an effect on CVD related endpoints.^7^, ^27^ Although not widely supported by actual data, it is plausible that the efficacy of statins is reduced in RTR as well.

RTR are a unique patient population due to their use of immunosuppressive medication. Standard immunosuppressive regimen includes a calcineurin inhibitor, either cyclosporine or tacrolimus, prednisolone and additional use of either the proliferation inhibitor mycophenolate mofetil (MMF), in case of RTR at high immunological risk, or an mTOR inhibitor.^28^ Cyclosporine has numerous dose‐dependent adverse effects, including nephrotoxicity and induction of haemolytic‐uremic syndrome.^29^ Cyclosporine is also known to increase CVD risk, at least partially attributable to increased circulating LDL particle numbers and increased oxidizability of LDL.^29^, ^30^, ^31^ Furthermore, increased homocysteine levels have been reported, as well as unfavourable effects on the fibrinolytic system.^32^ Use of cyclosporine is also associated with an elevated blood pressure, as well as increased risk of infections.^33^, ^34^, ^35^

Although the different statins share a similar mechanism of action they vary in their bioavailability, excretion and protein binding.^26^ Simvastatin, lovastatin and atorvastatin are metabolized in the liver primarily by cytochrome P450 3A4.^36^ Cyclosporine is also metabolized by cytochrome P450 3A4,^37^ making a bilateral pharmacological interaction plausible. And indeed, ample evidence shows that plasma levels of statins increase with concurrent administration of cyclosporine.^36^, ^38^, ^39^, ^40^, ^41^ However, a rise of statin plasma levels in cyclosporine treated patients was not only found in patients receiving statins metabolized by cytochrome P450 3A4, but as well in patients receiving fluvastatin, which is metabolized by cytochrome P450 2C9, therefore indicating that the mechanism of the interaction might not be restricted to competition at the level of the cytochrome P450 3A4 pathway.^37^ Interestingly, the increased systemic statin exposure does not lead to an increased lipid lowering effect.^38^ Furthermore, the incidence of myopathy was reported to be significantly higher with combined administration of cyclosporine and all statins except fluvastatin, ascribed to reduced clearance of statins and consequent higher serum concentrations.^42^, ^43^

On the other hand, less information is available about the effect of statins on cyclosporine levels. These are difficult to assess retrospectively as cyclosporine dosages are continuously adjusted based on serum levels. One study showed that both cyclosporine and pravastatin are excreted by P‐glycoprotein and multidrug‐resistant protein (MRP) 2 and that simultaneous administration of these drugs causes both a rise of cyclosporine in vivo,^44^ as well as pravastatin, due to competitive inhibition of the MRP2 transporter. It is therefore plausible to believe that simultaneous administration of cyclosporine and statins increases the bioavailability of both drugs due to inhibited metabolic clearance, therefore increasing the toxic and adverse cardiovascular effects of cyclosporine. Indeed, this is in line with our results, which show a higher risk on cardiovascular endpoints in cyclosporine treated patients.

Several limitations of our study need to be considered. It was carried out in a single centre, and analysis was conducted retrospectively. It is plausible that some form of prescription bias exists, despite propensity score matching. Further prospective research is warranted to validate our findings. Furthermore, although the study was sufficiently powered, the number of events was too small to be able to sub‐divide the primary CVD endpoint into different events. For the same reason, we were not able to assess the effect of different types of statins with sufficient certainty. Furthermore, the subgroup analysis of tacrolimus users consisted of a rather small number of subjects. Also, the studied population was predominantly Caucasian, creating difficulties in extrapolation of our findings to other ethnicities. Throughout recent years, a shift away from cyclosporine and towards tacrolimus as initial immunosuppressive regimen has taken place. However, in two groups use of cyclosporine is still favoured, namely in those where (i) treatment was initiated with cyclosporine and (ii) patients who are predisposed to tacrolimus related toxicity, such as new‐onset diabetes after transplantation (NODAT).^28^

In conclusion, to the best of our knowledge we are the first to evaluate the efficacy of stain use in RTR outside of the ALERT trial. Interestingly, our data indicate no obvious protective effect of statins with respect to lowering risk of CV events and CV mortality in RTR. Furthermore, statin use is potentially harmful in cyclosporine using RTR. The pathophysiological basis of these observations remains to be clarified, but it is plausible to assume a drug‐drug interaction of statins with cyclosporine, which leads to increased bioavailability of both drugs and a subsequent increase of adverse effects. Based on these data, we suggest that there is an apparent clinical need for prospective randomized controlled trials testing the impact of LDL‐C lowering with different therapeutic modalities on CVD outcomes in RTR.

## CONFLICT OF INTEREST

The authors of this manuscript have no conflicts of interest to disclose.

## AUTHOR CONTRIBUTIONS

JLCA: data acquisition and analysis, drafting the article and final approval for the version to be published; M.vd.G.: data analysis, critical article revision for important intellectual content and final approval for the version to be published; AWGN: data acquisition, critical article revision for important intellectual content and final approval for the version to be published; SJLB: data acquisition and analysis, critical article revision for important intellectual content and final approval for the version to be published; UJFT: conception and design of the study, interpretation of data, drafting the article and final approval of the version to be published.

## Supporting information

Supplementary Material
