# Supplementary material for: Statin use and incident cardiovascular events in renal transplant recipients
Source: Eur J Clin Invest. 2021 May 27;51(11):e13594. doi: 10.1111/eci.13594 (PMC8596424; doi:10.1111/eci.13594)
Supplement: Supplementary file 1 — Supplementary Material [file ECI-51-e13594-s001.docx]

**Supplementary Online Content**

**Statin use and incident cardiovascular events in renal transplant recipients**

Josephine L.C. Anderson^1^, Markus van der Giet^2^, Antonio W. Gomes Neto^1^, Stephan J.L. Bakker^1^, Uwe J.F. Tietge^1,3,4^

^1^Department of Internal Medicine, University Medical Center Groningen, University of Groningen, Groningen, The Netherlands

^2^Medizinische Klinik für Nephrologie und Internistische Intensivtherapie, Charité – Universitätsmedizin Berlin, 10117 Berlin, Germany

^3^Division of Clinical Chemistry, Department of Laboratory Medicine, Karolinska Institutet, Stockholm, Sweden

^4^Clinical Chemistry, Karolinska University Laboratory, Karolinska University Hospital, SE-141 86 Stockholm, Sweden

**Supplemental figure:** Propensity score histogram by treatment status.

**
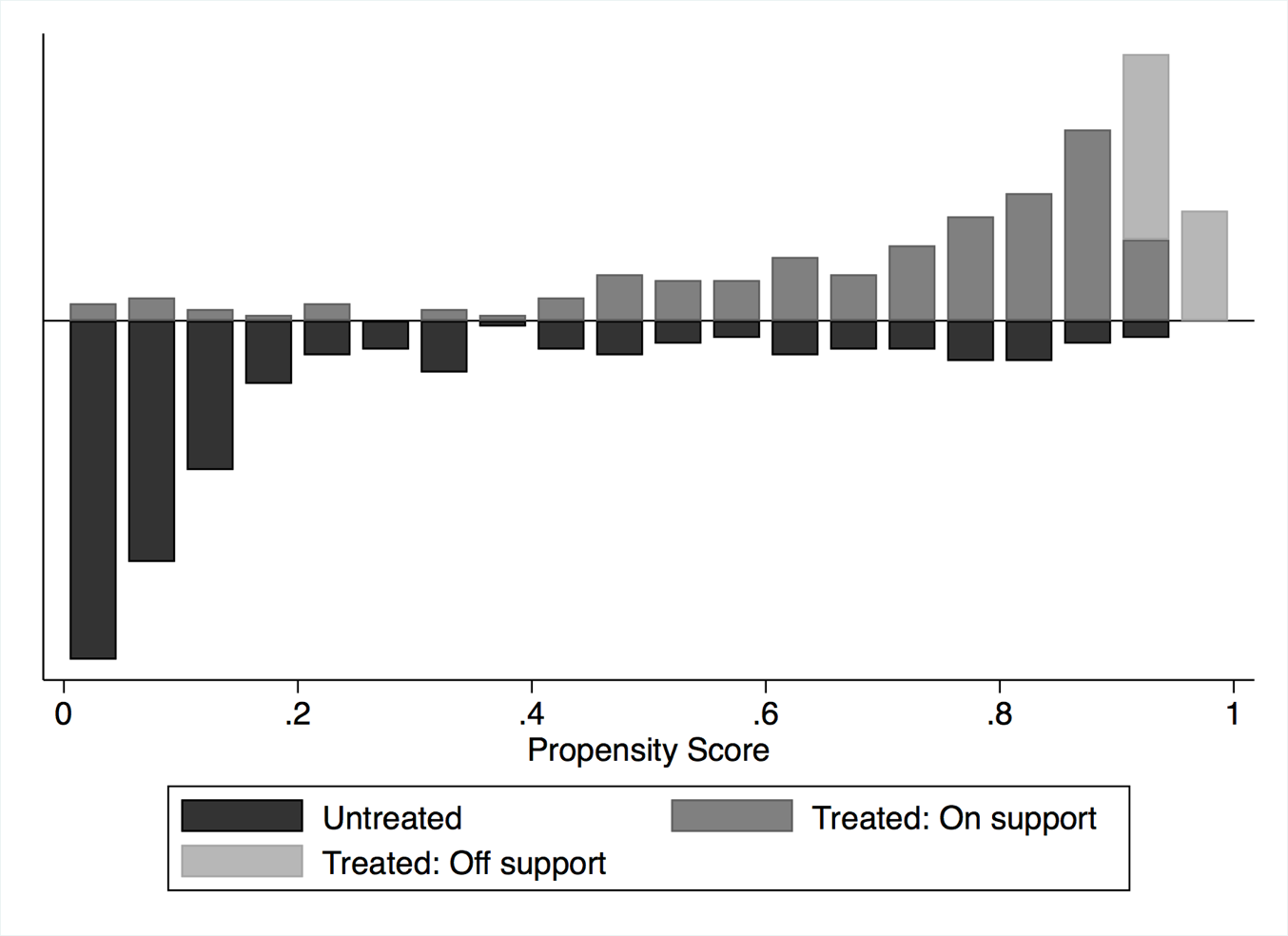
**

**Supplemental table.** Assessment of reduction of bias after propensity score matching.

| **Variable** | **Mean** | | | **t-test** | | **V(T)/V(C)** |
| --- | --- | --- | --- | --- | --- | --- |
|  | **Treated** | **Control** | **%bias** | **t** | **p** |  |
| Age | 57.577 | 56.246 | 11.0 | 1.36 | 0.174 | 1.01 |
| Gender | .42 | .368 | 10.5 | 1.19 | 0.235 | . |
| Serum creatinine | 136.41 | 140.23 | -6.5 | -0.75 | 0.455 | 0.80 |
| Primary renal disease | 225.21 | 225.73 | -3.4 | -0.40 | 0.693 | 1.16 |
| hsCRP | 2.452 | 2.33 | 4.1 | 0.56 | 0.578 | 1.46 |
| Triglycerides | 2.0335 | 2.0536 | -2.1 | -0.23 | 0.820 | 1.05 |
| Alcohol consumption | 1.276 | 1.356 | -11.8 | -1.25 | 0.210 | 0.99 |
| Dialysis time | 34.188 | 31.036 | 10.2 | 1.10 | 0.271 | 0.86 |
| Prednisone dose | 8.8654 | 8.975 | -5.8 | -0.71 | 0.477 | 1.51 |
| Diabetes Mellitus | .252 | .296 | 10.6 | 1.10 | 0.271 | . |
| Urinary protein excretion | .39705 | .72861 | -42.2 | -3.37 | 0.001 | 0.44 |
| Proliferation inhibitors | .828 | .808 | 5.4 | 0.58 | 0.563 | . |
| History of atherosclerotic CVD | .18 | .28 | -28.7 | -2.67 | 0.008 | . |
| Systolic blood pressure | 136.99 | 140.04 | -17.7 | -2.01 | 0.045 | 1.31 |
| BMI | 26.887 | 26.945 | -1.3 | -0.15 | 0.884 | 1.12 |
| Smoking | .78 | .932 | -22.6 | -2.39 | 0.017 | 0.76 |
| Number of antihypertensives | 1.616 | 1.652 | -5.2 | -0.69 | 0.488 | 1.31 |
| Use of cyclosporine | .42 | .308 | 22.9 | 2.62 | 0.009 | . |
| HDL | 1.4052 | 1.4296 | -5.1 | -0.50 | 0.618 | 0.52 |
| Glucose levels | 5.7156 | 5.8236 | -6.1 | -0.65 | 0.514 | 1.05 |
| HbA1c | 6.034 | 5.9432 | 11.7 | 1.52 | 0.128 | 1.48 |
| Metabolic syndrome | .764 | .756 | 1.8 | 0.21 | 0.835 | . |

Reduction of bias was assessed using a t-test for equality of means, the standardized percentage bias and the variance ratio. hsCRP, high sensitivity C-reactive protein; CVD, cardiovascular disease; BMI, body mass index; HDL, high density lipoprotein; HbA1c, Hemoglobin A1C.
